# Isolation of *Borrelia miyamotoi* and other Borreliae using a modified BSK medium

**DOI:** 10.1038/s41598-021-81252-1

**Published:** 2021-01-21

**Authors:** Adam J. Replogle, Christopher Sexton, John Young, Luke C. Kingry, Martin E. Schriefer, Marc Dolan, Tammi L. Johnson, Neeta P. Connally, Kerry A. Padgett, Jeannine M. Petersen

**Affiliations:** 1 Division of Vector-Borne Diseases, Centers for Disease Control and Prevention, Fort Collins, CO 80521 USA; 2 Department of Biological and Environmental Sciences, Western Connecticut State University, Danbury, CT 06810 USA; 3 California Department of Public Health, Infectious Diseases Branch/Vector-Borne Disease Section, Marina Bay Parkway, Richmond, CA 94804 USA; 4 Present Address: Texas A&M AgriLife Research, Uvalde, TX 78801 USA

**Keywords:** Microbiology, Bacteria, Clinical microbiology, Pathogens

## Abstract

*Borrelia* spirochetes are the causative agents of Lyme borreliosis (LB) and relapsing fever (RF). Despite the steady rise in infections and the identification of new species causing human illness over the last decade, isolation of borreliae in culture has become increasingly rare. A modified Barbour-Stoenner-Kelly (BSK) media formulation, BSK-R, was developed for isolation of the emerging RF pathogen, *Borrelia miyamotoi*. BSK-R is a diluted BSK-II derivative supplemented with Lebovitz’s L-15, mouse and fetal calf serum. Decreasing the concentration of CMRL 1066 and other components was essential for growth of North American *B. miyamotoi*. Sixteen *B. miyamotoi* isolates, originating from *Ixodes scapularis* ticks, rodent and human blood collected in the eastern and upper midwestern United States, were isolated and propagated to densities > 10^8^ spirochetes/mL. Growth of five other RF and ten different LB borreliae readily occurred in BSK-R. Additionally, primary culture recovery of 20 isolates of *Borrelia hermsii, Borrelia turicatae, Borrelia burgdorferi* and *Borrelia mayonii* was achieved in BSK-R using whole blood from infected patients. These data indicate this broadly encompassing borreliae media can aid in in vitro culture recovery of RF and LB spirochetes, including the direct isolation of new and emerging human pathogens.

## Introduction

Primary culture recovery is often considered the benchmark for proof of infection, but for many bacterial infections, including *Borrelia* spp., this proof is rarely achieved. Reasons for diminished use of culture for borreliae include their fastidious nature and strict requirement for exogenous nutrients and cofactors, lengthy in vitro doubling times, and the complex and expensive nature of culture media and its monitoring requirements^1,2^. Further, as infection can often be demonstrated by other indirect (e.g. serology) and direct (e.g. PCR) methods in a matter of hours, culture attempts may appear unnecessary and burdensome. Nevertheless, given the significant and growing public health importance of tickborne diseases^3,4^, including those caused by *Borrelia* species, the value of culture cannot be underestimated, especially as it pertains to conclusive demonstration of human infection in new or unexpected geographic regions and isolation of novel pathogens.

Human illness causing borreliae are divided into two genetically distinct groups, the *Borrelia burgdorferi *sensu lato (Bbsl) complex and the relapsing fever (RF) group^2^. Lyme borreliosis (LB), the most common tickborne illness in the northern hemisphere, results from infection by spirochetes in the Bbsl genospecies complex, which are transmitted through the bite of hard ticks (Ixodidae). Tickborne and louseborne RF develops upon infection by spirochetes in the RF group. Tickborne RF borreliae are transmitted by soft ticks (Argasidae); however, this paradigm shifted upon discovery of *Borrelia miyamotoi*^5^. Phylogenetic analysis places it within the RF borreliae, yet, *B. miyamotoi* is transmitted by *Ixodes* ticks^5^.

Historical attempts to propagate RF borreliae^6^ led to fundamental descriptions of their morphology, but it was not until 1971 that an in vitro culture media enabling primary recovery and continuous passage of the RF spirochete, *Borrelia hermsii*, was established^7^. Shortly thereafter (1983) the recovery and maintenance of a newly identified human pathogen, *Borrelia burgdorferi*, an agent of LB^8^, was described using a fortified version (Barbour-Stoenner-Kelly; BSK) of Stoenner-Kelly’s medium. In 1986, researchers from the Max von Pettenkofer Institute altered BSK media to culture *B. burgdorferi* in what has been referred to as modified Kelly-Pettenkofer (MKP) medium^9^. Today, primary culture recovery of borreliae, in both the RF and LB groups, is a more limited and specialized practice, perhaps even, a neglected laboratory tool. A standardized formulation, termed BSK-H^10^, is the only commercial formulation available.

Over the last decade several new *Borrelia* pathogens have emerged as causative agents of human illness. These include *Borrelia mayonii*, a genospecies within *B. burgdorferi *sensu lato, which was first described in 2016 as a novel cause of LB in the upper midwestern U.S.^11^. Infected patients demonstrate unusually high spirochetemia as compared to other *B. burgdorferi *sensu lato genospecies. *Borrelia miyamotoi*, first described in *Ixodes* ticks in Japan in 1995, was identified as a human pathogen in 2011 in Russia and 2015 in the United States^5,12,13^. Isolation of this spirochete from U.S. sources has proved challenging. Attempts to cultivate *B. miyamotoi* directly from *I. scapularis* and human CSF using BSK-H, were unsuccessful^13,14^.

A medium enabling direct isolation of species encompassing both the RF and LB borreliae groups may help to improve borreliae culture, while simultaneously facilitating isolation of new species. Here we describe the development of a modified BSK-II media formulation, BSK-R, which enabled the primary culture recovery of multiple U.S. *B. miyamotoi* isolates as well as other RF and LB borreliae, including *B. mayonii*.

## Results

### In vitro isolation of *B. miyamotoi* RI13-2395

**Figure 1:**
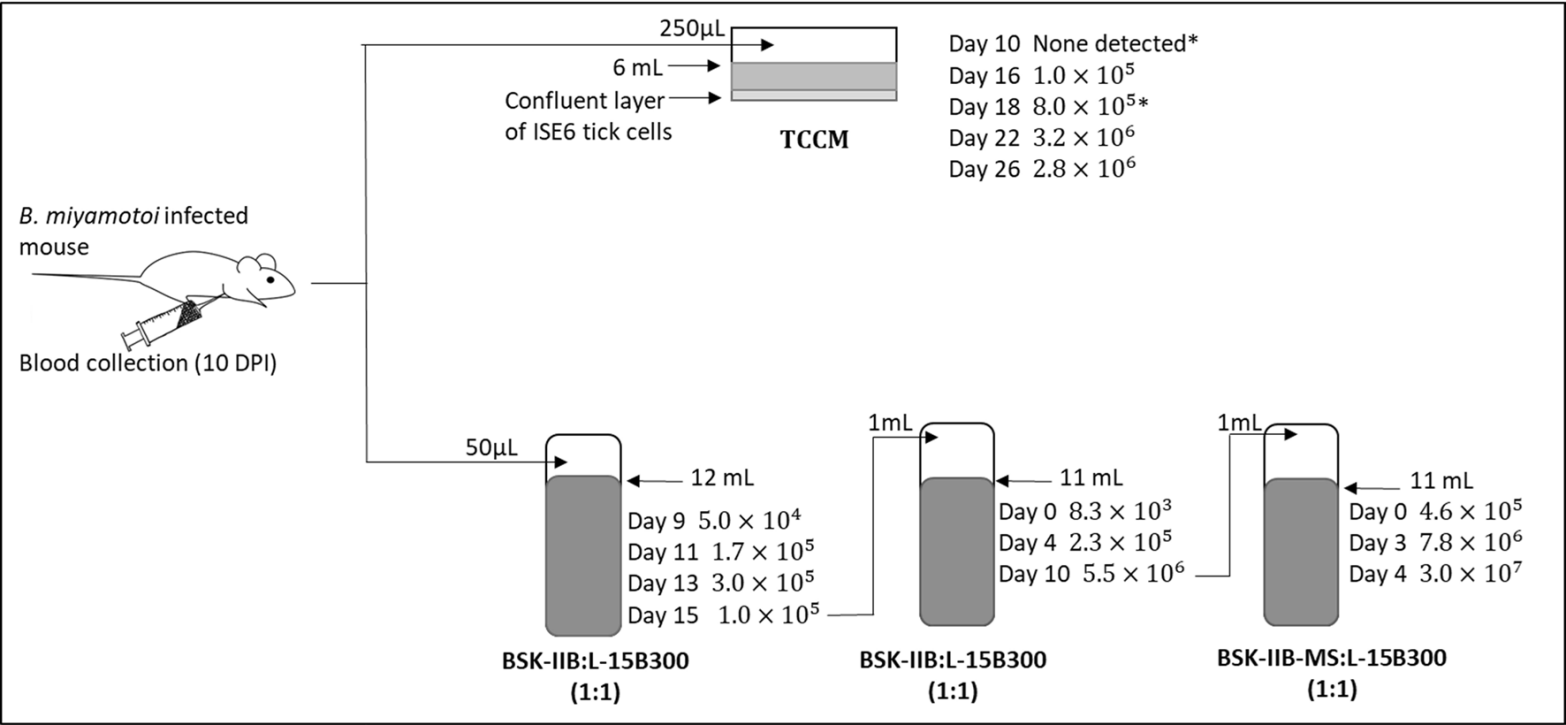
In vitro culture of North American *B. miyamotoi*. *I. scapularis* egg homogenate (*glpQ* PCR positive, *ospA* PCR negative) was inoculated subcutaneously into a BALB/c mouse. *Borrelia miyamotoi* DNA was detected in mouse blood on days six and ten post-infection; at the later time point, spirochetes were also visualized by dark field microscopy and confirmed as *B. miyamotoi* by 16S rDNA sequencing. On day ten, *B. miyamotoi* infected mouse blood was collected and used to seed liquid cultures, with (top) and without (bottom) ISE6 tick cells. Media compositions are shown. *Borrelia miyamotoi* concentrations are presented by day post-seeding of cultures. Concentrations shown are spirochetes/mL, as determined via counting of motile spirochetes by dark field microscopy. The * indicates timepoint at which TCCM was removed from tick cell co-cultures, centrifuged, and the resulting pellet resuspended in fresh TCCM and re-incubated with ISE6 cells. Day of passage into new media is shown by arrows (bottom); volumes used for passage are displayed.

In vitro isolation initially focused on *B. miyamotoi* from U.S. sources, given the prior lack of isolation in BSK-H. No growth occurred when blood from a laboratory infected mouse was seeded into BSK-IIB. In contrast, growth of *B. miyamotoi* occurred when the same blood was seeded into a confluent monolayer of ISE6 tick cells in tick cell co-culture medium (TCCM) as well as into BSK-IIB and L-15B300, the primary component of TCCM, combined at a ratio of 1:1 (Fig. 1). In the latter case, subsequent passage into BSK-IIB:L-15B300 (1:1), with BALB/c mouse serum substituted for rabbit serum (BSK-IIB-MS), yielded a peak spirochete density of 3.0 × 10^7^ spirochetes/mL. The recovered spirochetes were confirmed as *B. miyamotoi* by *glpQ* PCR and 16S rDNA sequencing (426 nucleotides, 100% identity to *B. miyamotoi* LB-2001). At this time, a strain name, RI13-2395, was assigned and either this actively growing culture or frozen stocks (passage 2; P2) used for subsequent experiments.

### Inhibition of *B. miyamotoi* growth in BSK-IIB

**Figure 2:**
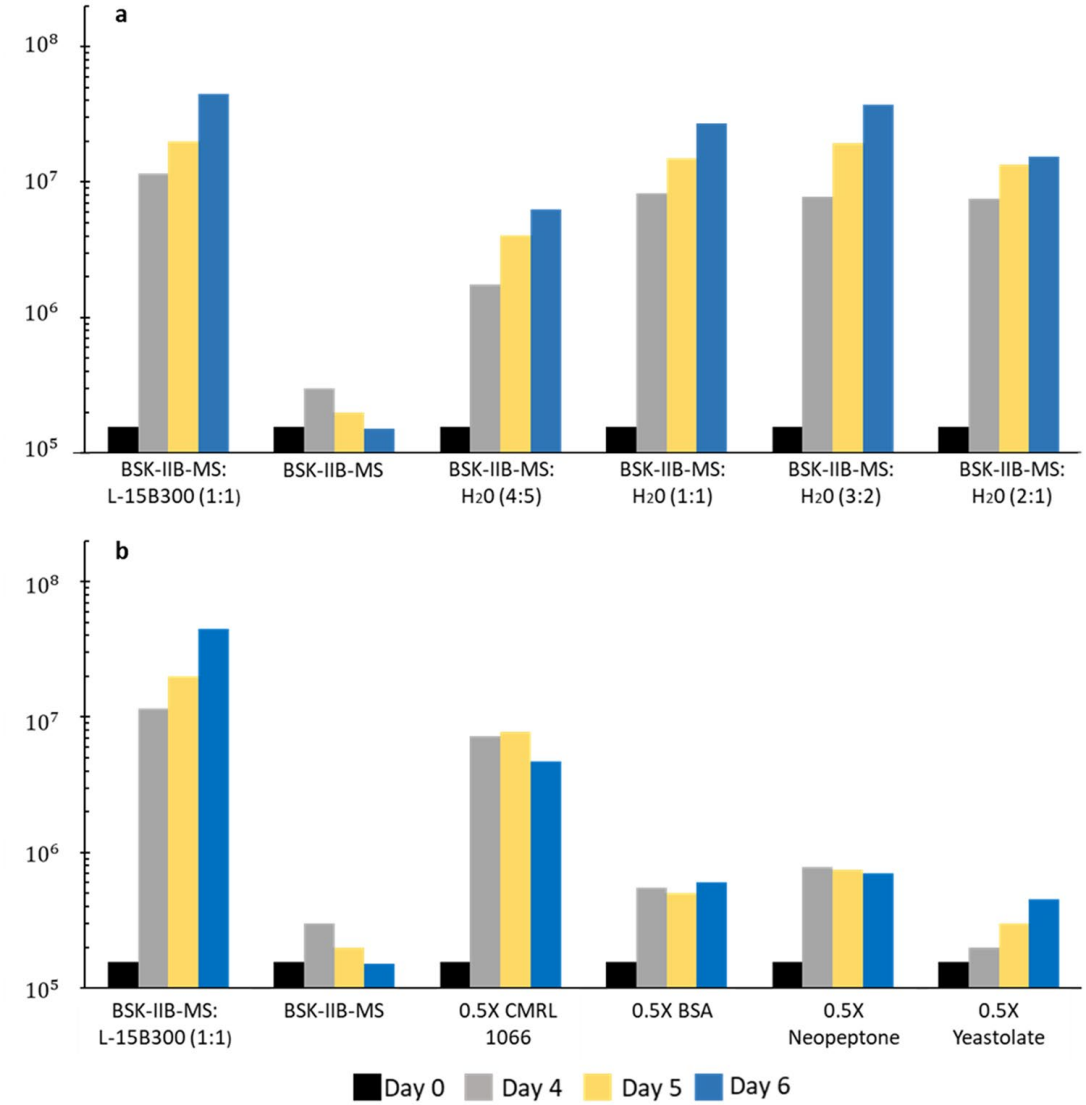
Inhibition of *B. miyamotoi* growth in BSK-IIB-MS. (**a**) Side-by-side comparisons of observed cell densities (spirochetes/mL) upon inoculation of BSK-IIB-MS:L-15B300 (1:1), BSK-IIB-MS alone and BSK-IIB-MS diluted with H_2_0 at the indicated ratios. Actively growing spirochetes were seeded into media formulations at a final concentration of 1. 6 × 10^5^ spirochetes/mL. The percentages of BSK-IIB-MS to H_2_0 in the 1:1 and 3:2 ratios, was 50:50 and 60:40, respectively. (**b**) Side by side comparisons of cell densities in BSK-IIB:L-15B300 (1:1), BSK-IIB-MS alone, and BSK-IIB-MS with individual indicated components reduced by half. The final concentration of indicated components was 4.04 g/L CMRL 1066 10X w/o L-glutamine, 20.85 g/L BSA, 2.01 g/L Bacto neopeptone and 0.84 g/L Bacto TC yeastolate. Actively growing spirochetes were seeded into media formulations at a final concentration of 1. 6 × 10^5^ spirochetes/mL. (**a**,**b**) Spirochete densities by day are indicated by color: day 0, black; day 4, grey; day 5, yellow; day 6, blue.

To determine if growth of *B. miyamotoi* RI13-2395 in BSK-IIB-MS:L-15B300 (1:1) was due to the addition of L-15B300 or from decreased concentrations of BSK-IIB ingredients, side-by-side growth comparisons were performed with BSK-IIB-MS:L-15B300 (1:1), BSK-IIB-MS alone, and BSK-IIB-MS mixed with H_2_0 at four different ratios (Fig. 2a). Actively growing spirochetes (P2) were inoculated into all media formulations at a final concentration of 1.6 × 10^5^ spirochetes/mL. Notably, no growth of *B. miyamotoi* occurred in BSK-IIB-MS. Only when BSK-IIB-MS was diluted with H_2_0 did growth of *B. miyamotoi* ensue. Similar growth (8 × 10^6^ spirochetes/mL; doubling time of 17 h) was achieved on day four for the 1:1,3:2 and 2:1 mixtures as compared to BSK-IIB-MS:L-15B300 (1:1) (10^7^ spirochetes/mL; doubling time of 16 h). The highest peak densities were observed when BSK-IIB-MS was diluted with H_2_0 at ratios of 1:1 and 3:2.

To narrow down which component/s of BSK-IIB-MS might be inhibitory, the final concentration of each of the ingredients, with the exception of serum and dithiothreitol (DTT), was singly reduced by half in BSK-IIB-MS. Decreasing the concentration of CMRL 1066 w/o L-glutamine by 50% (final concentration of 4.04 g/L) yielded a greater than 20-fold increase in spirochete density at day four post inoculation (doubling time of 17 h) (Fig. 2b). Individually reducing the concentration of proteinaceous components of BSK-IIB, Bacto neopeptone, bovine serum albumin (BSA), and Bacto TC yeastolate, and other components, by half did not relieve inhibition of *B. miyamotoi* growth to the same degree as was evidenced for CMRL 1066 (Fig. 2b; data not shown). Continuous growth of spirochetes was not observed beyond day four when any single component was reduced by half.

### BSK-R

BSK-IIB-MS diluted 40% with water [BSK-IIB-MS:H_2_O (3:2)] served as the basis for further media optimization using frozen P2 stocks of *B. miyamotoi* RI13-2395. Lebovitz’s L-15, a primary component of L-15B300, was added at final concentrations ranging from 4 to 10 g/L to look for growth augmentation. A final concentration of either 5 or 6.7 g/L [final concentration in BSK-IIB-MS:L-15B300 (1:1)] stimulated growth of *B. miyamotoi* by three-fold as compared to BSK-IIB-MS diluted 40% with water. A spirochete density of 3.0 × 10^7^ cells/mL was achieved after six days of growth when 5 g/L Lebovitz’s L-15 was included. At the uppermost concentration tested, 10 g/L, spirochete growth was impaired.

Table 1 provides a recipe for the final media formulation, designated BSK-R, which includes 5 g/L Leibovitz's L-15 and components of BSK-IIB adjusted to 60%, with the exception of 4.3 g/L CMRL-1066 w/o L-glutamine, 5.0 g/L glucose as well as 2.2 g/L HEPES and 0.98 g/L sodium bicarbonate, which were adjusted to give a final pH of 7.5. Serum was added to a final concentration of 13% (7% mouse serum, 4% rabbit serum and 2% fetal bovine serum).

**Table 1:** BSK media formulations.

BSK media components	BSK-IIB	BSK-IIB-MS	BSK-R
Probumin bovine serum albumin univeral grade, powder (Millipore Sigma, St. Louis, MA)	41.7 g/L	41.7 g/L	25 g/L
CMRL 1066 10X w/o L-glutamine (powder) (US Biological, Salem, MA)	8.08 g/L	8.08 g/L	4.3 g/L
Gibco Bacto neopeptone (Fisher Scientific, Pittsburgh, PA)	4.17 g/L	4.17 g/L	2.5 g/L
HEPES sodium salt, 99 + %, ACROS Organics (Fisher Scientific)	5.0 g/L	5.0 g/L	2.2 g/L
Sodium citrate dihydrate (Millipore Sigma)	0.583 g/L	0.583 g/L	0.35 g/L
D-( +)-Glucose (Millipore Sigma)	5.83 g/L	5.83 g/L	5.0 g/L
Sodium pyruvate (Millipore Sigma)	0.667 g/L	0.667 g/L	0.4 g/L
N-Acetyl-D-glucosamine, cell culture reagent (MP Biomedicals, Irvine, CA)	0.333 g/L	0.333 g/L	0.2 g/L
Sodium bicarbonate (Millipore Sigma)	1.83 g/L	1.83 g/L	0.98 g/L
Gibco Bacto TC yeastolate (Fisher Scientific)	1.67 g/L	1.67 g/L	1.0 g/L
L-cysteine hydrochloride monohydrate (Fisher Scientific)	0.233 g/L	0.233 g/L	0.14 g/L
Mouse BALB/c serum (Innovative Research, Novi, MI)	None	12%	7%
Rabbit serum, sterile, trace-hemolyzed (Pel-Freez, Rogers, AR)	12%	None	4%
Corning premium fetal bovine serum (Fisher Scientific)	None	None	2%
Invitrogen UltraPure dithiothreitol (Fisher Scientific)	0.067 g/L	0.067 g/L	0.04 g/L
Gibco Leibovitz's L-15 medium, powder (Fisher Scientific)	None	None	5.0 g/L

Comparison of *B. miyamotoi* growth in BSK-R, BSK-IIB-MS diluted 40% with water, and BSK-IIB-MS:L-15B300 (1:1), using a P2 frozen stock as the starting inoculum, indicated that *B. miyamotoi* strain RI13-2395 displayed an improved doubling time of 18 h in BSK-R as compared to 23 h in the original BSK-IIB-MS:L-15B300 (1:1) formulation used initially to propagate this strain. The spirochete density achieved at this time point (96 h) was also two-fold higher in BSK-R as compared to BSK-IIB-MS:L-15B300 (1:1). After eight days, a maximum density was reached, with 1.3 × 10^8^, 3.9 × 10^7^ and 7.2 × 10^7^ spirochetes/mL observed in BSK-R, BSK-IIB-MS diluted 40% with water and BSK-IIB-MS: L-15B300 (1:1), respectively.

### Direct isolation of *B. miyamotoi* from *Ixodes scapularis *ticks and *Tamias striatus* in BSK-R

Seventeen single nymphs, derived from five female adult *B. miyamotoi* infected *I. scapularis* ticks, were seeded into BSK-R with antibiotics. *Borrelia miyamotoi* spirochetes were successfully recovered in 14 of the 17 inoculated cultures, yielding a recovery rate of 82%. The average density without passage was 1.1 × 10^7^ spirochetes/mL and the average time to this density was 11.5 days (range seven to 14 days). Spirochetes were confirmed as *B. miyamotoi* by *glpQ* PCR and/or 16S rDNA sequencing. Additionally, one isolate, CT13-2396, was genome sequenced, with this sequence made publicly available (GenBank accession numbers CP017126 to CP017140)^15^*.* When blood (collected with EDTA) from a field collected chipmunk (*T. striatus)* was inoculated into BSK-R with antibiotics, growth also occurred, albeit at slower rates as compared to *I. scapularis* ticks. Motile spirochetes (1 × 10^4^ spirochetes/mL) were observed after 13 days. Upon passage of spirochetes to fresh BSK-R, a density of 7.7 × 10^6^ spirochetes was achieved 11 days later. The recovered spirochete was *ospA* PCR negative and *glpQ* PCR positive.

No growth of *B. miyamotoi* occurred in BSK-R when nine single minced *I. pacificus* nymphs, derived from a field collected female adult tick (*glpQ* PCR positive, *ospA* PCR negative), were incubated for 30 days, including two blind passages into fresh media. Seeding of blood (20 µL) from a SCID mouse infected with *B. miyamotoi* via *I. pacificus* feeding, also yielded no growth in BSK-R, however, motile spirochetes (3.5 × 10^4^ spirochetes/mL) were detected microscopically in ISE6 co-cultures and subsequently passaged into BSK-R. The recovered *B. miyamotoi* isolate, CA17-2241, was genome sequenced with the chromosome and large linear plasmid sequences publicly available (GenBank accession numbers CP021872 and CP021873)^16^.

### BSK-R supports growth of both relapsing fever and Lyme borreliae

Side-by-side comparisons demonstrated three other RF borreliae, *B. hermsii*, *Borrelia turicatae,* and *Borrelia recurrentis*, grew to high densities without passage in BSK-R and BSK-IIB, with ~ 10^8^ spirochetes/mL achieved for all (Table 2). Two *B. burgdorferi *sensu lato genospecies, *B. burgdorferi* and *B. mayonii,* were similarly propagated in both BSK-R and BSK-IIB (Table 2). As expected, the *B. miyamotoi* isolates recovered in this study, LB-2001 and three *B. miyamotoi* strains originating from Japan all grew in BSK-R with maximum concentrations ranging from 3.6 × 10^7^ to 1.5 × 10^8^ spirochetes/mL after 6–10 days. In contrast, BSK-IIB was unable to support growth of any North American *B. miyamotoi* strain, with the exception of *B. miyamotoi* CA17-2241, which reached a maximum cell density of 4.2 × 10^6^ spirochetes/mL after 19 days. For the three *B. miyamotoi* strains originating from Japan, spirochete motility and growth rate were improved in BSK-R as compared to BSK-IIB. Twelve additional strains encompassing *Borrelia parkeri*, *Borrelia coriaceae*, *Borrelia afzelii*, *Borrelia garinii*, *Borrelia bissettii*, *Borrelia lanei*, *Borrelia valasiana*, *Borrelia yangtzensis*, *Borrelia sinica* and *Borrelia spielmanii* all readily proliferated in BSK-R, without passage, to peak motile spirochete densities of 10^7^ to 10^8^ spirochetes/mL (data not shown).

**Table 2:** Growth comparison of Lyme and relapsing fever *Borrelia* species in BSK-R and BSK-IIB.

*Borrelia* group	*Borrelia* species	Geographic origin	BSK-IIB growth	BSK-IIB maximum density	BSK-R growth	BSK-R maximum density
Relapsing fever	*B. miyamotoi* RI13-2395	United States (RI)	No	NA	Yes	1.5 × 10^8^
	*B. miyamotoi* CT13-2396	United States (CT)	No	NA	Yes	1.4 × 10^8^
	*B. miyamotoi* CT15-0838	United States (CT)	No	NA	Yes	1.2 × 10^8^
	*B. miyamotoi* CT15-0839	United States (CT)	No	NA	Yes	1.2 × 10^8^
	*B. miyamotoi* CT15-0840	United States (CT)	No	NA	Yes	1.3 × 10^8^
	*B. miyamotoi* CT15-0841	United States (CT)	No	NA	Yes	1.5 × 10^8^
	*B. miyamotoi* MN16-2304	United States (MN)	No	NA	Yes	6.8 × 10^7^
	*B. miyamotoi* CA17-2241	United States (CA)	Yes	4.2 × 10^6^	Yes	6.2 × 10^7^
	*B. miyamotoi* LB2001	United States (CT)	No	NA	Yes	3.6 × 10^7^
	*B. miyamotoi* HT31	Japan	Yes	9.0 × 10^7^	Yes	7.0 × 10^7^
	*B. miyamotoi* HT24	Japan	Yes	9.0 × 10^7^	Yes	1.5 × 10^8^
	*B. miyamotoi* FR64b	Japan	Yes	8.5 × 10^7^	Yes	1.3 × 10^8^
	*B. hermsii* NE95-0544	United States (CO)	Yes	1.8 × 10^8^	Yes	8.5 × 10^7^
	*B. turicatae* TX15-4645	United States (TX)	Yes	2.0 × 10^8^	Yes	2.0 × 10^8^
	*B. recurrentis* SU99-0699	Republic of Sudan	Yes	1.5 × 10^8^	Yes	1.0 × 10^8^
Lyme	*B. burgdorferi* B31	United States (NY)	Yes	2.9 × 10^8^	Yes	1.5 × 10^8^
	*B. mayonii* MN14-1420	United States (MN)	Yes	2.3 × 10^7^	Yes	1.7 × 10^8^
	*B. mayonii* MN14-1539	United States (MN)	Yes	1.5 × 10^7^	Yes	1.7 × 10^8^

### Direct isolation of *Borrelia* species from human blood and field collected ticks using BSK-R

When 21 human whole bloods (50–500 µL collected with EDTA) from infected patients were inoculated into BSK-R with antibiotics, isolates of both RF (*B. miyamotoi, B. hermsii, B. turicatae)* and Lyme borreliae (*B. mayonii* and *B. burgdorferi*) were successfully recovered (Table 3). For all five species, spirochetes were detected by periodic microscopy after three to 15 days incubation without passage. A single *B. mayonii* culture required blind-passage on day eight into fresh BSK-R at a dilution of 1% (v/v) for detection of spirochetes; this sample was not received until 38 days post collection with considerable hemolysis evident. In all cases, recovered spirochetes were subsequently propagated to densities ≥ 5 × 10^6^ spirochetes/mL. Starting inocula were estimated at < 10^3^ to 10^7^ spirochetes based on microscopic quantitation of the original blood samples. A microscopic limit of detection of 8.3 × 10^3^ spirochetes/mL blood (one spirochete in 600 fields of blood diluted 1:10) precluded determination of spirochete numbers lower than 10^3^. The average time for microscopic detection of spirochetes in *B. hermsii, B. turicatae* and *B. mayonii* recovered cultures was six, seven, and nine days, respectively. The single *B. miyamotoi* isolate required 15 days of growth for detection. The quality of the blood specimen (hemolysis, contamination, length of time from collection to culture), the level of spirochetemia and the infecting *Borrelia* species were associated with the time to microscopic detection of spirochetes in culture. Lower inocula and low-quality specimens correlated with longer recovery times. One *B. hermsii* isolate was successfully recovered from blood collected two days after azithromycin treatment, while the *B. burgdorferi* isolate was recovered from a heavily contaminated blood specimen. All recovered isolates were verified to species level by PCR, eight housekeeping gene multi-locus sequence typing or 16S sequencing.

**Table 3:** Primary culture recovery of Lyme and relapsing fever *Borrelia* species in BSK-R.

*Borrelia* group	*Borrelia* species	Length of time (days) between blood collection and culture	Concentration of spirochetes per mL blood based on microscopy	Motile spirochetes (%) in blood	Blood volume inoculated (µL)	No. of spirochetes inoculated	Time to detection of spirochetes in BSK-R: concentration (spirochetes/mL)	References
Relapsing fever	*B. miyamotoi*	8	< 8.2 × 10^3^	NA	500	< 4.1 × 10^3^	Day 15: 2.0 × 10^4^	Unpublished
	*B. hermsii*	4	2.5 × 10^5^	100%	50	1.3 × 10^4^	Day 4: 8.2 × 10^3^	^37^
	*B. hermsii*	4	2.5 × 10^4^	100%	50	1.3 × 10^3^	Day 8: 1.5 × 10^5^	^37^
	*B. hermsii*	4	2.5 × 10^4^	100%	50	1.3 × 10^3^	Day 8: 3.5 × 10^5^	^37^
	*B. hermsii*	4	2.5 × 10^4^	100%	50	1.3 × 10^3^	Day 4: 5.0 × 10^4^	^37^
	*B. hermsii*	6	2.9 × 10^6^	100%	50	1.4 × 10^5^	Day 3: 6.0 × 10^4^	Unpublished
	*B. hermsii*	5	< 2.5 × 10^4^	NA	100	< 2.5 × 10^3^	Day 7: 6.7 × 10^3^	Unpublished
	*B. hermsii*	7	8.8 × 10^7^	1%	100	8.8 × 10^6^	Day 5: 7.1 × 10^6^	Unpublished
	*B. hermsii*	5	2.0 × 10^5^	100%	500	1.0 × 10^5^	Day 7: 1.0 × 10^4^	Unpublished
	*B. hermsii*^a^	9	1.5 × 10^5^	17%	500	7.5 × 10^4^	Day 6: 1.5 × 10^4^	Unpublished
	*B. hermsii*	3	3.6 × 10^6^	83%	500	1.8 × 10^6^	Day 5: 1.7 × 10^7^	Unpublished
	*B. hermsii*	22	1.1 × 10^7^	0%	500	5.5 × 10^6^	Day 8: 1.6 × 10^8^	Unpublished
	*B. hermsii*	17	3.0 × 10^7^	0%	500	1.5 × 10^7^	Day 7: 9.3 × 10^6^	Unpublished
	*B. turicatae*	6	< 4.9 × 10^4^	NA	100	< 4.9 × 10^3^	Day 7: 3.5 × 10^7^	^38,39^
	*B. turicatae*	9	< 8.2 × 10^3^	NA	500	< 4.1 × 10^3^	Day 7: 5.0 × 10^4^	^34^
	*B. turicatae*	7	< 8.2 × 10^3^	NA	500	< 4.1 × 10^3^	Day 7: 5.6 × 10^7^	^34^
Lyme	*B. mayonii*	5	1.5 × 10^5^	100%	50	< 7.5 × 10^3^	Day 6: 1.5 × 10^6^	^11,35,40^
	*B. mayonii*	39	< 2.5 × 10^4^	NA	50	< 1.3 × 10^3^	Day 16: 9 × 10^5b^	^11,40^
	*B. mayonii*	8	< 8.2 × 10^3^	NA	500	< 4.1 × 10^3^	Day 7: 1 × 10^5^	Unpublished
	*B. mayonii*	Unknown	8.0 × 10^3^	50%	500	< 4.0 × 10^3^	Day 7: 4 × 10^6^	Unpublished
	*B. burgdorferi*	17	< 8.2 × 10^3^	NA	500	< 4.1 × 10^3^	Day 13 = 1.7 × 10^5^	Unpublished

^a^Blood draw 2 days post azythromycin treatment.

^b^Blind passaged after 8 days.

Direct recovery of *B. burgdorferi* and *B. miyamotoi* was also achieved when single field-collected *I. scapularis* adult ticks (n = 55) were homogenized and inoculated into BSK-R with antibiotics. Twenty-six isolates of *B. burgdorferi* were readily obtained within seven days of growth and confirmed by PCR (*ospA* positive, *glpQ* negative). Markedly, both *B. burgdorferi* and *B. miyamotoi* were simultaneously recovered in BSK-R within seven days of inoculating two co-infected ticks. Concurrent culture recovery of both *B. burgdorferi* and *B. miyamotoi* was confirmed by PCR (*ospA* and *glpQ* positive) and sequencing.

## Discussion

BSK-R, a diluted BSK-II derivative supplemented with Lebovitz’s L-15, mouse and fetal calf serum, was developed and successfully used for the direct in vitro isolation of 15 freezer-retrievable isolates of *B. miyamotoi* originating from animal and tick sources in the eastern or upper midwestern U.S. This media also broadly supported growth of 15 other *Borrelia* species encompassing the RF group as well as the LB group, all of which grew from frozen stocks to unpassaged cell densities of > 10^7^ spirochetes/mL. Notably, primary culture*s* (21 isolates) of *B. miyamotoi*, *B. hermsii, B. turicatae, B. burgdorferi* and *B. mayonii* were successfully recovered from the blood of infected patients using BSK-R. These include the first isolations from human cases in the U.S. for the two emerging pathogens, *B. miyamotoi* (this report) and *B. mayonii*^11^.

Unexpectedly, we discovered North American *B. miyamotoi* only grew in BSK-IIB-MS when all components were diluted with water. This finding yielded the single most important media modification for successful growth of *B. miyamotoi*. The primary component responsible for growth inhibition was CMRL 1066. Decreasing its final concentration by 50% to 4.04 g/L, with all other components held constant, enabled initial growth of *B. miyamotoi*, although it was not sufficient for sustained propagation. The concentration of motile bacteria arrested at day four, suggesting at least one other component of BSK-IIB-MS also negatively impacts *B. miyamotoi* growth. CMRL 1066 was originally incorporated into Stoenner’s enrichment of Kelly’s medium [final concentration estimated to be 4.87 mg/mL (5% of a 10X solution)] to improve antigen expression and growth of *B. hermsii*^17^. The concentration of CMRL 1066 in BSK-II, which was optimized for growth of *B. burgdorfer*i, is ~ 7.1 mg/mL^18^. The concentration of CMRL 1066 in BSK-H is ~ 10 mg/mL. We postulate the higher concentration of CMRL 1066 and other components is why *B. miyamotoi* from ticks and patients in the U.S. was not cultivable in this medium^13,14^. Although Lebovitz’s L-15 augmented growth of *B. miyamotoi*, it was not able to substitute for CMRL 1066 (data not shown). Lebovitz’s L-15, like CMRL 1066, is comprised of amino acids and vitamins, but without other factors present in CMRL 1066. It is buffered with salts, free base amino acids and galactose to help maintain physiological pH control in CO_2_ free systems^19^.

The sensitivity of *Borrelia* species to serum complement varies by animal source and has been linked to reservoir host competency in nature^20^. Initial incorporation of mouse serum into media formulations was based on evidence suggesting *B. miyamotoi* is resistant to mouse serum complement. *Borrelia miyamotoi* was initially detected in mice in nature^5^. Additionally, mouse serum (CD1 and BALB/c) was not bactericidal for *B. miyamotoi* when tested in an in vitro assay, whereas rabbit serum was (data not shown). The negative effects of rabbit sera on *B. miyamotoi* could be decreased, but not eliminated, when serum was inactivated at 0.5 h at 56 °C. The observed sensitivity to rabbit serum complement is consistent with a prior study where 50% human serum, but not rabbit serum, was suitable for propagation of *B. miyamotoi*^21^. Further inactivation of rabbit serum for 45 min and incorporation into BSK-R as the sole source of serum (13%) was found to support growth of *B. miyamotoi*, albeit with lower spirochete motility and slower growth as compared to mouse serum. Nonetheless, this finding indicates rabbit serum, a less expensive alternative to mouse serum, can substitute when using BSK-R for propagation of *Borrelia* stock cultures. Continued work is needed to further define serum requirements for the direct in vitro recovery of North American *B. miyamotoi*.

Primary culture recovery of *Borrelia* species in BSK-R was successfully achieved from bloods, of infected patients, collected in EDTA and with spirochete concentrations estimated at < 10^3^ and of low quality (collected 39 days prior, no motile spirochetes, heavily contaminated, and post-antibiotic treatment). Initially, blood was seeded into BSK-R at dilutions no higher than 1% (v/v), with no removal of blood components. Subsequently, this protocol was modified to include removal of EDTA and components released from hemolyzed blood cells, given their inhibitory effect on growth of *B. miyamotoi* and/or *B. burgdorferi*^22–24^. With this modification, larger blood volumes were successfully used for recovery of cultures.

The time to detection of *B. miyamotoi* in primary cultures was slowed and the unpassaged spirochete density lower when blood specimens versus tick homogenates were used as the starting inoculum. The two isolates recovered from animal and human blood both required ~ 14 days of growth in BSK-R for their detection, whereas the recovery time from tick homogenates was as low as seven days, even when *B. burgdorferi* was concurrently isolated. This suggests something about the inocula themselves, such as a higher spirochete number in ticks, an inhibitory component in blood or a growth component in ticks, might be responsible for this difference. PCR quantitation has been used to estimate the number of *B. miyamotoi* genome equivalents in blood from infected U.S. patients at 10^4^–10^5^/mL^25^; whether the number of spirochetes in *I. scapularis* ticks is higher requires further investigation. Of note, primary culture recovery of *B. miyamotoi* from the plasma of patients in Russia also required incubation in MKP-F for 12–20 days^22^. The slowed time for primary recovery of *B. miyamotoi* is consistent with its longer doubling time as compared to other borreliae. A doubling time of ~ 18 h was determined for U.S. *B. miyamotoi* in BSK-R and in MKP supplemented with 50% human serum^21^, whereas the doubling time for *B. mayonii* in BSK-R is ~ 9 h. Given primary isolation of *B. miyamotoi* from blood requires ~ 14 days, it’s likely media components critical for growth deteriorate with age and why growth could be improved upon detection of spirochetes and transfer into fresh media.

An important consideration for *Borrelia* culture is whether the recovered isolate retains pathogenicity and infectivity. As shown previously for *B. burgdorferi*, differences in medium composition can significantly affect downstream experiments, due to the loss of plasmids^26^. Towards this end, *B. miyamotoi* and *B. mayonii* strains isolated in primary culture, using BSK-R, were used to successfully establish infection in CD1 or SCID mice via subcutaneous infection^27–33^. *Borrelia mayonii* infected CD1 mice also supported infection of *I. scapularis* via feeding^32^. Prior publications referred to BSK-R as either modified BSK or modified BSK-II^11,15,16,27–40^.

*Borrelia miyamotoi* was not isolated from *I. pacificus* in BSK-R, although it was successfully propagated in BSK-R, once an isolate was recovered via co-culture of infected SCID mouse blood with an embryonic tick cell line, ISE6. The reason for this is not clear and requires further work. Challenges occurred with transmission and feeding of *I. pacificus* on both immunocompetent and SCID mice and may have been due to the condition of the ticks. Mold was present in the maintained ticks and few spirochetes were observed in tick midguts. As sequences of *B. miyamotoi* from *I. pacificus* and *I. scapularis* have been shown to differ, it is also possible strain differences might be associated with recovery in BSK-R^41^.

While this work was in progress, two tick-derived isolations of North American *B. miyamotoi* were reported. M1029 and LB-2001 were isolated from SCID mouse plasma in MKP diluted with 50% human serum and in MKP-F (which is diluted with 10% fetal calf serum), respectively^21,42^. We postulate that growth in these two cases is due to a reduced concentration of CMRL 1066; 3.2 and 6.3 g/L for MKP containing 50% human serum and MKP-F, respectively, as well as other components. In the case of MKP supplemented with 50% human serum, the volume of serum added would also have reduced the concentrations of all other MKP ingredients by half^21^. While the addition of 50% human serum likely had a positive effect on growth, our finding that *B. miyamotoi* grew in BSK-IIB-MS diluted 50% with water, suggests the reduction of MKP components by half may be the primary reason for growth of North American *B. miyamotoi* in this media.

Given the lengthy turnaround times and specialized growth requirements of *Borrelia* species, use of culture as a standard diagnostic test is limited. Nonetheless, its value should not be understated. Isolation of *Borrelia* species is paramount to a better understanding of the natural biology and pathogenesis of newly identified human pathogens or those which represent distinct geographic and ecological niches. Additionally, the public health importance of *Borrelia* species dictates the use of culture as a diagnostic benchmark for a true understanding of the geographic regions these infections occur. The development of a media formulation that enables growth and direct isolation of both RF and LB *Borrelia* species, should help to improve our understanding of these important tick-transmitted pathogens. Given that batch/lot differences of BSK ingredients have previously been shown to influence spirochete recovery^10^ further research directed towards optimization and standardization of BSK-R as well as simplification is important.

## Materials and methods

### Specimen sources

Adult *I. scapularis* were collected in South Kingstown, Rhode Island and Danbury, Ridgefield, and Newtown, Connecticut. Adult *I. pacificus* were collected in Napa, Marin, Sonoma, and Santa Clara, California. Adult ticks were fed on New Zealand White rabbits (Harlan, Indianapolis, IN). Post-ovaposition females were tested by TaqMan PCR for *B. burgdorferi* and *B. miyamotoi*. *Borrelia miyamotoi* PCR positive egg clutches were incubated in desiccators at 21 °C and > 97% RH in Wheaton vials (Millville, NJ) containing plaster of Paris and activated charcoal and allowed to hatch to larvae. Larvae (5–100 per mouse) were allowed to feed to repletion on CD1 mice and molt to nymphs. Blood was collected in EDTA tubes from small mammals live trapped in Minnesota^35^. All work with laboratory mice was approved by the Centers for Disease Control (CDC) and Prevention Division of Vector-Borne Diseases Animal Care and Use Committee in accordance with the Animal Welfare Act. Human EDTA whole blood specimens were submitted for *Borrelia* diagnostic testing or were residual samples from research studies*.*

### Mouse infection

Approximately 200 *I. scapularis* eggs, were surface sterilized by submerging in H_2_O_2_ for one minute, rinsed with sterile saline, submerged in 70% ethanol for 2 min, rinsed again and ground in a 1.7 mL Eppendorf tube containing 200 µLs sterile saline using a plastic pestle. BALB/c mice were inoculated subcutaneously with 100 µLs of egg homogenate. To infect SCID mice, *I. pacificus* nymphal ticks (3 per mouse) were allowed to feed to repletion. Mice were euthanized and blood collected by cardiac puncture without the use of anticoagulant.

### Tick cell co-culture

*I. scapularis* embryonic (ISE6) cells^43^ were cultivated in tissue culture flasks with plug seal caps at 34 °C with caps tight. Tick Cell Co-culture Media (TCCM) consisted of 90% L-15B300, prepared according to Munderloh^44^ and 10% BSK-IIB, a modification of the media used to culture *Borrelia lonestari*^45^. For seeding confluent cell cultures, 20–250 µL of infected mouse blood was added to flasks. Media was replaced every 7–10 days, by decanting, followed by centrifugation at 3300(g) for 8 min. The supernatant was discarded, the resultant pellet resuspended in 5–6 mL fresh media and transferred back into the flask.

### Culture in BSK formulations

For development of BSK-R, early passage (P2) non-frozen and frozen stocks (− 80 °C) of *B. miyamotoi* strain RI13-2395 were used. Frozen stocks were prepared in cryovials by adding glycerol to a final concentration of 18% and placing into cryo 1 °C freezing containers (Thermo Fisher, Waltham, MA) at − 80 °C for 24 h. Frozen stocks were warmed at − 20 °C for approximately 30 min followed by placing at 4 °C or in ice bucket until thawed (~ 15–30 min). For side-by-side media comparisons, multiple stocks were thawed, combined into a single tube and mixed by inverting. For direct comparison of BSK-R and BSK-IIB media formulations, frozen (− 80 °C) strains were thawed and mixed, and the same volume simultaneously inoculated into both media. Cultures were followed by microscopy until an unpassaged peak density for motile spirochetes was achieved.

Frozen aliquots of media were used for all experiments unless indicated. For preparation of BSK-IIB-MS formulations combined with H_2_0, filtered MilliQ H_2_0 was added at the indicated ratio to complete BSK-IIB-MS media. For preparation of BSK-IIB-MS with single ingredients reduced by half, the final concentration of all other ingredients remained unchanged; media was freshly prepared and not frozen.

For inoculation of BSK-R, 50 µL of EDTA whole blood from field collected animals was added, followed by gentle mixing via pipetting. Red blood cells (RBCs) were allowed to settle for 48 h and the supernatant transferred to a new tube. EDTA was not removed. *Ixodes* ticks were surface sterilized by 3% H_2_O_2_ (5 min) and 70% ethanol (11 min) washes and air dried on bibulous paper. Nymphs minced with scalpels on glass slides and adults homogenized using Wheaton Tenbroeck tissue grinders (DWK Life Sciences Millville, NJ) were inoculated into cultures. 50–500 µLs of EDTA whole blood from patients was used to seed cultures. If blood volumes larger than 100 µLs were inoculated, red blood cells (RBCs) were allowed to settle overnight. To remove EDTA and red blood cell components, the resulting supernatant was removed and centrifuged at 3300(g) for 8 min, with the pellet resuspended in fresh media.

All cultures were incubated in 5 mL or 14 mL snap cap round bottom polypropylene Falcon tubes or 5 mL Eppendorf centrifuge tubes at 34 °C with tubes filled to 90% capacity and caps pressed on tight, creating a microaerophilic environment. BSK-R seeded with blood from infected animals or patients was refreshed after approximately two weeks of culture; supernatants were removed, and centrifuged at 3,300(g) for 8 min, with the pellet resuspended in fresh media. Tubes were examined periodically by dark field microscopy for spirochete growth.

### Cultures

The Japanese strains of *B. miyamotoi*, HT31, HT24 and FR64b, were kindly provided by Dr. Fukunaga via Dr. Barbara Johnson. Strain LB2001 was provided by Dr. Joppe Hovius (The Academic Medical Center in the Department of Internal Medicine, Division of Infectious Diseases). *Borrelia bissettii* (DSM 17990)*, B. valaisiana* (DSM 21467)*, B. spielmanii* (DSM 16813)*, B. sinica* (DSM 23262)*, B. yangtzensis* (DSM 24625), and *B. lanei* (DSM 17992) were obtained from DSMZ. *Borrelia hermsii* (NE95-0544)*, B. mayonii* (MN14-1420, MN14-1539), *B. burgdorferi* (B31), *B. parkeri* (MT90-0900)*, B. coriaceae* (89-1425), *B. turicatae* (TX15-4645), and *B. recurrentis* (SU99-0699) were from the CDC reference collection.

### Microscopy

Wet mounts for dark field microscopy were prepared by pipetting 5 µLs of culture or a 1:10 dilution of whole blood onto a glass slide and covering with a 22 mm × 22 mm coverslip. Wet mounts were visualized using an Axio Imager.A2 microscope (Ziess, Thornwood, NY) at a total magnification of 400X. The number of spirochetes per field was determined by counting motile spirochetes in at least 20 fields. The exception was for human blood specimens, where both motile and non-motile spirochetes were counted. The number of spirochetes per mL was determined as follows: average number of spirochetes per 400X field × (5 × 10^5^ spirochetes/mL) dilution factor. The conversion factor was determined as previously described^11^. Spirochete motility was assessed based on observation of rapid corkscrewing movement.

### DNA extraction, PCR and sequencing

Female ticks or egg clutches (~ 50–400) were placed in a MagNA Lyser green bead tube (Roche, Indianapolis, IN) with tissue lysis buffer (Roche) and homogenized in a MagNA Lyser (Roche) at 6500 rpm for 50 s, once for eggs, three times for adults, and DNA extracted using a MagNA Pure 96 (Roche). DNA from mouse blood and cultures was extracted using the QIAamp DNA Mini kit (Qiagen, Valencia, CA).

*Borrelia miyamotoi glpQ* primers and probe were modified from Ullmann et al.^46^: BmglpQ-F GACAATATTCCTGTTATAATGC, BmglpQ-R CACTGAGATTTAGTGATTTAAGTTC, and BmglpQ-P FAM-CCCAGAAATTGACACAACCAC-BHQ1. The *B. burgdorferi ospA* primers were modified from Ivacic et al.^47^: OspA-F AATATTTATTGGGAATAGGTCTAA, OspA-R CACCAGGCAAATCTACTGA and TTAATAGCATGTAAGCAAAATGTTAGCA-BHQ1. Each 20 µL PCR reaction contained 5 mM MgCl_2_, 500 nM each primer, 100 nM probe, 1 × PerfeCta qPCR FastMix II Low ROX (Quanta Biosciences, Beverly, MA), 5 µL DNA extract. Cycling conditions on the 7500 FAST Dx (Applied Biosystems, Foster City, CA) were 95 °C 3 min, and 50 cycles of 95 °C for 10 s and 57 °C for 30 s. PCR assays for *B. turicatae* and *B. hermsii* were as described^48^. For sequencing, primers were glpQ-F GGAGCTGACTACCTAGAAC, glpQ-R GGGTATCCAAGGTCCAATTCC, 16 s-F CCTGGCTTAGAACTAACG, and 16 s-R TTCGCCTCTGGTATTCTTCC. Reactions were as previously described^34^. Consensus sequences were assembled with Clone Manager 9.3 (Sci-Ed, Denver, CO) and aligned to *Borrelia* sequences in GenBank. One to eight housekeeping genes were amplified, sequenced, and analyzed for identification of *B. mayonii*^25^.

### Media formulations

To prepare 600 mL of an in-house formulation for *B. hermsii* growth, (here termed BSK-IIB), 500 mL Milli-Q water and 25.0 g universal grade Bovine Serum Albumin (BSA) (Millipore Sigma, St. Louis, MO) was added to a 1 L flask and stirred slowly until fully dissolved. The following reagents were next added, one at a time, ensuring each reagent was fully dissolved before addition of others; 4.85 g CMRL 1066 10X w/o L-glutamine, powder, (US Biological, Salem, MA), 2.5 g Gibco Bacto neopeptone (Fisher Scientific, Pittsburgh, PA), 3.0 g HEPES sodium salt (Fisher Scientific), 0.35 g sodium citrate (Millipore Sigma), 3.5 g D-( +)-Glucose (Millipore Sigma), 0.4 g sodium pyruvate, sodium salt (Millipore Sigma), 0.2 g N-acetyl-D-glucosamine (MP Biomedicals, Irvine, CA), 1.1 g sodium bicarbonate, anhydrous (Millipore Sigma), 1.0 g Gibco Bacto TC yeastolate (Fisher Scientific), and 0.14 g tissue culture grade L-cysteine HCl monohydrate (Fisher Scientific). Once all reagents were fully dissolved, 72 mL of heat inactivated (56 °C for 45 min) sterile, trace hemolyzed rabbit serum (Pel-Freez, Rogers, AR) was added followed by 0.04 g Invitrogen UltraPure dithiothreitol (DTT) (Fisher Scientific). Finally, the pH was adjusted to 7.5 and antibiotics were added followed by filter sterilization using a 0.22 µm Millipore Express PLUS filtration system (Millipore Sigma). This same formulation with substitution of heat inactivated (56 °C for 30 min) BALB/c mouse serum (Innovative Research, Novi, MI) in place of rabbit serum was termed BSK-IIB-MS.

To prepare 600 mL of BSK-R, 300 mL Milli-Q water and 15.0 g BSA (universal grade) was added to a 1 L flask and stirred slowly until fully dissolved. Simultaneously, 200 mL Milli-Q water and 3.0 g Gibco Leibovitz's L-15 medium, powder (Fisher Scientific) was added to a 500 mL flask and stirred slowly until fully dissolved. To the 1 L flask containing fully dissolved BSA, the following reagents were added one at a time ensuring each reagent was fully dissolved before addition of next one; 2.6 g CMRL 1066 10X w/o L-glutamine (powder), 1.5 g Gibco Bacto neopeptone, 1.3 g HEPES sodium salt, 0.21 g sodium citrate, 3.0 g D-( +)-Glucose, 0.24 g sodium pyruvate, sodium salt, 0.12 g N-acetyl-D-glucosamine, 0.59 g sodium bicarbonate, 0.6 g Gibco Bacto TC yeastolate and 0.084 g L-cysteine HCl monohydrate. After reagents were fully dissolved, 24 mL heat inactivated (56 °C for 45 min) trace hemolyzed, sterile rabbit serum, 42 mL heat inactivated (56 °C for 30 min) BALB/c mouse serum and 12 mL heat inactivated (56 °C for 30 min) Corning premium fetal bovine serum (FBS) (Fisher Scientific) were added to the same flask, followed by 0.024 g Invitrogen UltraPure DTT and the fully dissolved Leibovitz’s L-15 medium. Finally, the pH was adjusted to 7.5, antibiotics were added and the media filter sterilized as above.

The final concentrations of antibiotics included 0.05 g/L rifampicin (Fisher Scientific), 0.02 g/L cycloheximide (Fisher Scientific), 0.2 g/L phosphomycin (MP Biomedicals), and 0.0025 g/L (6 mL of a 250 µg/mL solution) amphotericin B (Fisher Scientific).

Single use aliquots of complete media (including serum and antibiotics) were prepared in 5 mL or 14 mL falcon tubes and stored at − 20 °C in the dark for up to one year.

### Ethics statement

The secondary use of residual samples described herein was performed in accordance with the relevant guidelines and regulations under a protocol approved by the CDC Institutional Review Board with an approved waiver of informed consent. The views and opinions expressed herein are those of the authors alone and do not represent the official position of the Centers for Disease Control and Prevention.
